# Novel Pyran-Linked Phthalazinone-Pyrazole Hybrids: Synthesis, Cytotoxicity Evaluation, Molecular Modeling, and Descriptor Studies

**DOI:** 10.3389/fchem.2021.666573

**Published:** 2021-05-24

**Authors:** M. Shaheer Malik, Basim H. Asghar, Riyaz Syed, Reem I. Alsantali, Moataz Morad, Hatem M. Altass, Ziad Moussa, Ismail I. Althagafi, Rabab S. Jassas, Saleh A. Ahmed

**Affiliations:** ^1^ Department of Chemistry, Faculty of Applied Sciences, Umm Al-Qura University, Makkah, Saudi Arabia; ^2^ Department of Chemistry, Jawaharlal Nehru Technological University, Hyderabad, India; ^3^ Department of Pharmaceutical Chemistry, Pharmacy College, Taif University, Makkah, Saudi Arabia; ^4^ Research Laboratories Unit, Faculty of Applied Science, Umm Al-Qura University, Makkah, Saudi Arabia; ^5^ Department of Chemistry, College of Science, United Arab Emirates University, Al Ain, United Arab Emirates; ^6^ Department of Chemistry, Jamoum University College, Umm Al-Qura University, Makkah, Saudi Arabia; ^7^ Department of Chemistry, Faculty of Science, Assiut University, Assiut, Egypt

**Keywords:** phthalazinone, pyrazole hybrids, pyran, multicomponent, anticancer activity, molecular modelling, molecular descriptors

## Abstract

A series of novel pyran-linked phthalazinone-pyrazole hybrids were designed and synthesized by a facile one-pot three-component reaction employing substituted phthalazinone, 1H-pyrazole-5-carbaldehyde, and active methylene compounds. Optimization studies led to the identification of L-proline and ethanol as efficient catalyst and solvent, respectively. This was followed by evaluation of anticancer activity against solid tumor cell lines of lung and cervical carcinoma that displayed IC_50_ values in the range of 9.8–41.6 µM. Molecular modeling studies were performed, and crucial interactions with the target protein were identified. The drug likeliness nature of the compounds and molecular descriptors such as molecular flexibility, complexity, and shape index were also calculated to understand the potential of the synthesized molecules to act as lead-like molecule upon further detailed biological investigations as well as 3D-QSAR studies.

## Introduction

**FIGURE 1:**
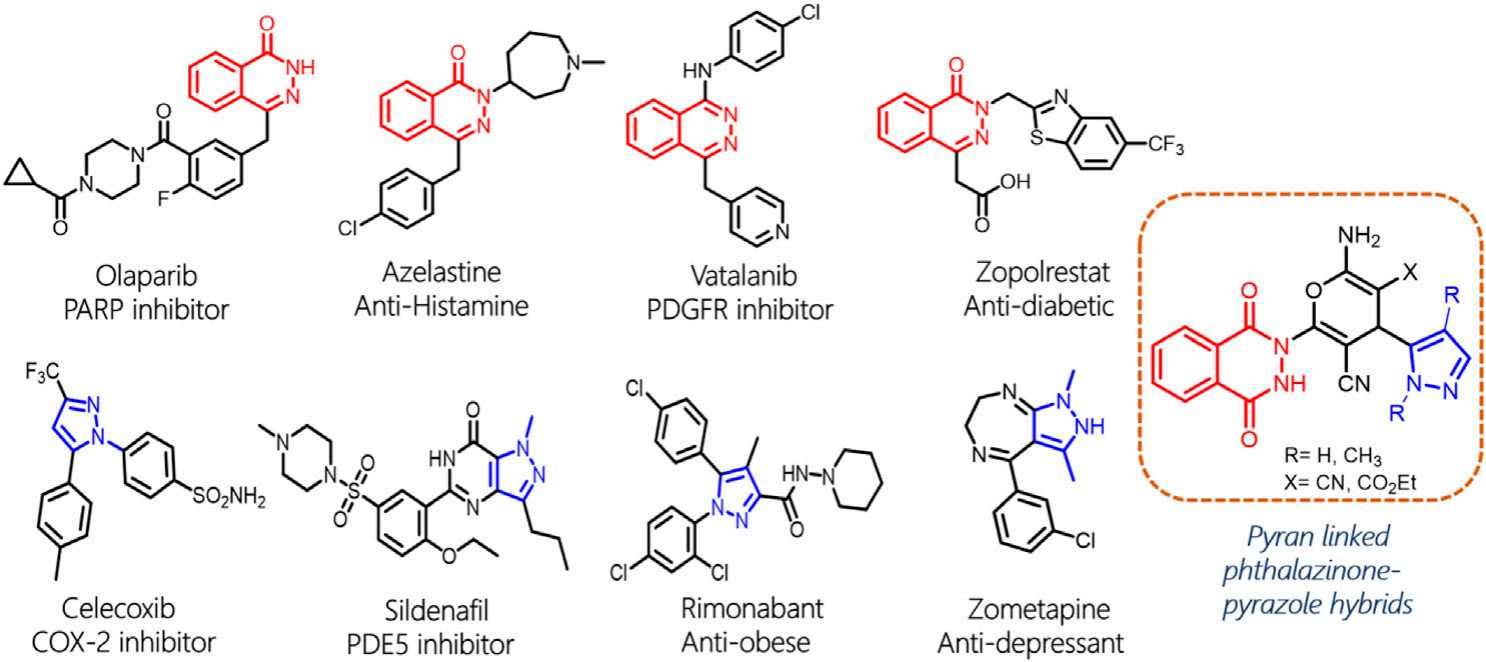
Drugs with phthalazine or pyrazole moieties and design strategy of phthalazine pyrazoles.

Phthalazine and its derivative phthalazinone are heterocyclic scaffolds that are extensively explored for potential application in the treatment of a wide range of medical conditions (Vila et al., 2015). Given its significance as a pharmacophore, phthalazine is a key moiety in various new chemical entities that exhibit antidiabetic, anticonvulsant, anti-inflammatory, antihypertensive, and analgesic activities (Young Taek et al., 2017; Sangshetti et al., 2019; Mohd and Mohammad, 2020). Particularly, a wide array of phthalazine-derived compounds have been reported as anticancer agents for various molecular targets (Sung Kim et al., 2004; Loh et al., 2005; Wang et al., 2014). Further, the importance of phthalazine is highlighted by the presence of such pharmacophore as a part of several marketed drugs such as olaparib, azelastine, vatalanib, and zopolrestat (Figure 1; Inskeep et al., 1994; Joensuu et al., 2011; Gunderson and Moore, 2015; Cheng et al., 2019). Moreover, phthalazines also exhibit promising prospects as fluorescence probes and luminescence materials (Saha et al., 2013; Martins et al., 2019). Similarly, the pyrazole molecule, a five-membered heterocycle with two nitrogen atoms, has emerged as a powerful scaffold due to its therapeutic potential. Pyrazole scaffold–containing chemical agents have a broad spectrum of biological activity and are well documented to display a plethora of pharmacological properties, such as antiviral, antibacterial, anti-HIV, anti-fungicidal, antidepressant, antidiabetic, antitumor, anti-inflammatory, and antihelminthic activities (Khan et al., 2016; Karrouchi et al., 2018; Bennani et al., 2020). Additionally, the pyrazole scaffold is a cornerstone in various marketed drugs such as celecoxib, sildenafil, rimonabant, and zometapine that are widely used for different therapeutic indications (Boyd and Fremming, 2005; Krasselt and Baerwald, 2019; Krishnappa et al., 2019).

The advent of green and efficient multicomponent reactions (MCRs) offers immense opportunity to explore new chemical reactions, which could generate vital pharmacophores in a facile and eco-friendly way (Isambert and Lavilla, 2008; Dömling et al., 2012; Rotstein et al., 2014). In MCRs, multiple bonds are formed between three or more reactants in a single step with environmental benignity and greener aspects such as atom economy, less solvent consumption, and reduced waste generation. Some other added advantages are improved yields, higher selectivity, facile construction of complex molecules, and no need for isolation of intermediates (Cioc et al., 2014). In recent years, the research endeavors focused on developing new MCRs, and several synthetic protocols have been reported in this direction (Jethava et al., 2020; Wang et al., 2021). The amalgamation of MCRs with a naturally occurring catalyst renders a more environmental-friendly chemical methodology. The naturally occurring amino acid, L-proline, and its derivatives are prime organocatalysts with a wide application profile in asymmetric organic synthesis (Sarita et al., 2016; Liu and Wang, 2017).

In continuation of our research endeavors in the development of novel anticancer agents (Malik et al., 2019; Malik et al., 2021) and considering the pharmaceutical significance of two heterocyclic scaffolds, phthalazine and pyrazole, we envisaged to tether these scaffolds in a single chemical entity with a pyran ring as the linker. Interestingly, the pyran moiety itself exhibits immense pharmaceutical potential such as anticancer, antiviral, diuretic, and other properties (Kumar et al., 2017). On the chemical synthesis front, the multicomponent reactions, catalyzed by environmental-friendly catalysts, need to be harnessed to access novel chemical compounds with improved sustainability and lowered environmental burden. Herein, we report the synthesis of novel pyran-linked phthalazinone-pyrazole hybrids by one-pot three-component reactions by using L-proline as a catalyst. The synthesized compounds were screened for their anticancer activity on selected cancer cell lines of lung and cervix cancers. The active hybrids were docked to understand their interaction with human serine hydroxymethyltransferase 2 (SHMT2), a protein that is upregulated in lung and other cancer cells. Finally, the drug likeliness properties and molecular descriptors are computationally calculated to identify molecules for further development.

## Materials and Methods

Reactions were monitored by thin-layer chromatography (silica gel glass plates containing 60 F-254), and TLC plates were visualized by UV light or iodine indicator. Infrared (IR) spectra were recorded on VERTEX 70 Brucker by using KBr. NMR spectra were recorded on a Bruker DRX-400 spectrometer, and chemical shifts were reported in ppm, downfield from internal TMS standard. Mass spectra were recorded on Agilent-LCMS instrument. Starting materials and reagents were procured commercially or synthesized in the laboratory.

### Chemical Synthesis

General procedure for the synthesis of pyran-linked phthalazinone-pyrazole hybrids **4a**–**h**:

3-(1,4-dioxo-3,4-dihydrophthalazin-2(1H)-yl)-3-oxopropanenitrile (**1**) (1 equiv, 2.29 g, 10 mmol), 1H-pyrazole-5-carbaldehyde (**2a**–**2d**) (1 equiv, 10 mmol), and malononitrile **3a** (1equiv, 0.66 g, 10 mmol) or ethyl 2-cyanoacetate **3b** (1eq, 1.13 g, 10 mmol) were added into ethanol (50 ml) in the presence of 20 mol% L-proline (0.23 g) as a catalyst and heated at 70–75°C for 50–60 min. The progress of the reaction was monitored by TLC. After completion of the reaction, the reaction mixture was cooled to 30–35°C, and cold water was added to the reaction mixture and stirred for 30 min. The resulting solid was separated through direct filtration to afford crude form of **4a**–**h**. Finally, the product was recrystallized from ethanol and dried at 60–65°C for 10–12 h to obtain pure form of **4a**–**h**.

2-Amino-6-(1,4-dioxo-3,4-dihydrophthalazin-2(1H)-yl)-4-(1H-pyrazol-5-yl)-4H-pyran-3,5-dicarbonitrile (**4a**): Mp 250–252°C; IR (KBr) cm^−1^: 3,184–3,544 (-NH-), 2,191 (-CN-), 1,739 (-CO-); ^1^H-NMR (DMSO-d_6_, 400 MHz): δ 6.5 (s, 1H, -CH), 7.4 (d, 1H, *J* = 7.8 Hz, Ar-H), 7.9–8.1 (m, 4H, Ar-H), 8.2 (d, 1H, *J* = 7.5, Ar-H), 9.8 (s, 2H, -NH_2_), 11.4 (s, 1H, -NH), 12.2 (s, 1H, -NH); ^13^C NMR (DMSO-d_6_, 100 MHz): δ 48.7, 75.1, 87.2, 113.9, 115.8, 124.2, 125.8, 128.6, 129.5, 131.7, 134.3, 137.1, 155.8, 157.1, 163.3, 163.5 [M + H^+^]: 374 (see Supplementary Material).

2-Amino-6-(1,4-dioxo-3,4-dihydrophthalazin-2(1H)-yl)-4-(1-methyl-1H-pyrazol-5-yl)-4H-pyran-3,5-dicarbonitrile (**4b**): Mp: 242–244°C; IR (KBr) cm^−1^: 3,093–3,519 (-NH-), 2,258 (-CN-), 1748 (-CO-); ^1^H-NMR (DMSO-d_6_, 400 MHz): δ 2.9 (s, 3H, -CH_3_), 6.5 (s, 1H, -CH), 7.4 (d, 1H, *J* = 7.8 Hz, Ar-H), 7.9–8.1 (m, 4H, Ar-H), 8.1 (d, 1H, *J* = 7.5 Hz, Ar-H), 9.6 (s, 2H, -NH_2_), 12.0 (s, 1H, -NH); ^13^C NMR (DMSO-d_6_, 100 MHz): δ 28.8, 48.5, 75.4, 86.7, 114.5, 115.0, 124.5, 127.9, 128.6, 129.7, 130.8, 134.1, 136.0, 155.7, 157.4, 163.0, 163.6 [M + H^+^]: 388.

2-Amino-6-(1,4-dioxo-3,4-dihydrophthalazin-2(1H)-yl)-4-(4-methyl-1H-pyrazol-5-yl)-4H-pyran-3,5-dicarbonitrile (**4c**): Mp: 238–240°C; IR (KBr) cm^−1^: 3,137–3,584 (-NH-), 2,252 (-CN-), 1742 (-CO-); ^1^H-NMR (DMSO-d_6_, 400 MHz): δ 3.4 (s, 3H, -CH_3_), 6.4 (s, 1H, -CH), 7.9–8.1 (m, 4H, Ar-H), 8.2 (d, 1H, *J* = 7.5, Ar-H), 9.8 (s, 2H, -NH_2_), 11.3 (s, 1H, -NH), 12.1 (s, 1H, -NH); ^13^C NMR (DMSO-d_6_, 100 MHz): δ 34.0, 49.4, 74.2, 84.5, 115.2, 116.5, 123.4, 126.8, 128.9, 129.9, 131.7, 133.9, 136.2, 154.8, 157.6, 162.1, 163.3 [M + H^+^]: 388.

2-Amino-6-(1,4-dioxo-3,4-dihydrophthalazin-2(1H)-yl)-4-(1H-pyrazol-4-yl)-4H-pyran-3,5-dicarbonitrile (**4d**): Mp: 254–256°C IR (KBr) cm^−1^: 3,038–3,586 (-NH-), 2,256 (-CN-), 1745 (-CO-); ^1^H-NMR (DMSO-d_6_, 400 MHz): δ 6.4 (s, 1H, -CH), 7.4–8.2 (m, 6H, Ar-H), 9.9 (s, 2H, -NH_2_), 11.3 (s, 1H, -NH),12.2 (s, 1H, -NH); ^13^C NMR (DMSO-d_6_, 100 MHz): δ 46.6, 74.5, 86.3, 114.6, 115.9, 125.5, 127.7, 129.2, 129.9, 131.6, 134.4, 137.5, 155.2, 157.4, 163.5, 163.9 [M + H^+^]: 374.

Ethyl 2-amino-5-cyano-6-(1,4-dioxo-3,4-dihydrophthalazin-2(1H)-yl)-4-(1H-pyrazol-5-yl)-4H-pyran-3-carboxylate (**4e**): Mp: 234–236°C; IR (KBr) cm^−1^: 3,159–3,401 (-NH-), 2,218 (-CN-), 1724 (-CO-); ^1^H-NMR (DMSO-d_6_, 400 MHz): δ 1.2 (t, 3H, *J* = 6.8 Hz, -CH_3_), 3.0 (q, 2H, *J* = 7.6 Hz, -CH_2_), 6.6 (s, 1H, -CH), 7.4 (d, 1H, *J* = 7.4 Hz, Ar-H), 7.5 (t, 2H, *J* = 7.6 Hz, Ar-H), 7.6 (t, 2H, *J* = 7.5 Hz, Ar-H), 8.0 (d, 1H, *J* = 7.4 Hz, Ar-H), 9.6 (s, 2H, -NH_2_), 11.5 (s, 1H, -NH), 13.0 (s, 1H, -NH); ^13^C NMR (DMSO-d_6_, 100 MHz): δ 14.0, 41.2, 58.1, 76.7, 88.1, 111.2, 113.6, 115.7, 117.9, 118.9, 122.2, 124.9, 126.0, 127.3, 128.8, 131.3, 137.1, 141.1, 158.7, 167.7, 167.7; [M + H^+^]: 421.

Ethyl 2-amino-5-cyano-6-(1,4-dioxo-3,4-dihydrophthalazin-2(1H)-yl)-4-(1-methyl-1H-pyrazol-5-yl)-4H-pyran-3-carboxylate (**4f**): Mp: 243–245°C; IR (KBr) cm^−1^: 3,037–3,581 (-NH-), 2,257 (-CN-), 1748 (-CO-); ^1^H-NMR (DMSO-d_6_, 400 MHz): δ 1.2 (t, 3H, *J* = 6.8 Hz, -CH_3_), 2.8 (s, 3H, -CH_3_), 3.0 (q, 2H, *J* = 7.6 Hz, -CH_2_), 6.6 (s, 1H, -CH), 7.3 (d, 1H, *J* = 7.3 Hz, Ar-H), 7.5 (t, 2H, *J* = 7.6 Hz, Ar-H), 7.6 (t, 2H, *J* = 7.6 Hz, Ar-H), 8.0 (d, 1H, *J* = 7.5 Hz, Ar-H), 9.6 (s, 2H, -NH_2_), 13.0 (s, 1H, -NH); ^13^C NMR (DMSO-d_6_, 100 MHz): δ 14.1, 19.9, 36.4, 42.4, 58.1, 76.7, 88.1, 112.4, 112.8, 115.7, 118.0, 118.8, 123.0, 125.2, 126.0, 128.1, 128.8, 131.3, 139.1, 141.8, 159.7, 167.1, 167.4 [M + H^+^]: 435.

Ethyl 2-amino-5-cyano-6-(1,4-dioxo-3,4-dihydrophthalazin-2(1H)-yl)-4-(4-methyl-1H-pyrazol-5-yl)-4H-pyran-3-carboxylate (**4g**): Mp: 256–258°C; IR (KBr) cm^−1^: 3,138–3,482 (-NH-), 2,156 (-CN-), 1732 (-CO-); ^1^H-NMR (DMSO-d_6_, 400 MHz): δ 1.1 (t, 3H, *J* = 6.8 Hz, -CH_3_), 2.9 (s, 3H, -CH_3_), 3.1 (q, 2H, *J* = 7.4 Hz, -CH_2_), 6.5 (s, 1H, -CH), 7.5 (t, 2H, *J* = 7.4 Hz, Ar-H), 7.6 (t, 2H, *J* = 7.6 Hz, Ar-H), 8.1 (d, 1H, *J* = 7.4 Hz, Ar-H), 9.5 (s, 2H, -NH_2_), 13.1 (s, 1H, -NH); ^13^C NMR (DMSO-d_6_, 100 MHz): δ 14.2, 18.9, 36.3, 41.5, 58.2, 76.6, 88.4, 111.3, 112.9, 114.8, 118.1, 118.9, 123.1, 124.1, 125.8, 128.2, 128.9, 131.5, 139.4, 141.9, 159.8, 167.5, 167.9 [M + H^+^]: 435.

Ethyl 2-amino-5-cyano-6-(1,4-dioxo-3,4-dihydrophthalazin-2(1H)-yl)-4-(1H-pyrazol-4-yl)-4H-pyran-3-carboxylate (**4h**): Mp: 253–255°C; IR (KBr) cm^−1^: 3,136–3,482 (-NH-), 2,256 (-CN-), 1743 (-CO-); ^1^H-NMR (DMSO-d_6_, 400 MHz): δ 1.1 (t, 3H, *J* = 6.8 Hz, -CH_3_), 3.1 (q, 2H, *J* = 7.4 Hz, -CH_2_), 6.5 (s, 1H, -CH), 7.2 (s, 1H, Ar-H), 7.4 (t, 2H, *J* = 7.5 Hz, Ar-H), 7.6 (t, 2H, *J* = 7.6 Hz, Ar-H), 8.1 (s, 1H, Ar-H), 9.5 (s, 2H, -NH_2_), 11.4 (s, 1H, -NH), 13.1 (s, 1H, -NH); ^13^C NMR (DMSO-d_6_, 100 MHz): δ 14.1, 41.4, 57.3, 75.8, 89.3, 111.4, 113.7, 115.8, 117.8, 118.5, 121.4, 124.5, 126.2, 127.5, 128.8, 131.4, 137.2, 141.4, 158.6, 167.6, 167.9 [M + H^+^]: 421.

### Cytotoxicity Assay

The cell viability of A549 (human lung carcinoma) and Hela (human cervical carcinoma) cells was evaluated by using the MTT colorimetric assay (Sigma, United States) (Mosmann, 1983). The cancer cells were seeded at a density of 2 × 10^4^ cells in 100 µL of cell culture medium (DMEM containing 10% fetal bovine serum, 100 μg/ml streptomycin, and 100 μg/ml penicillin) per well of 96-well plate and grown for a period of 24 h prior to the addition of the test compounds. Cells were incubated with different concentrations of test compounds for 48 h. After incubation, the wells were washed with 200 µL of PBS, and then 10% MTT solution was added to each well and incubated for 2 h at 37°C. The formazan crystals were solubilized by the addition of 100 µL of DMSO, and then the optical density was recorded at 570 nm using a multimode reader (Tecan Infinite 200 PRO, Switzerland). Each experiment was repeated three times (*n* = 3), and the results are specified as mean with standard deviation.

### Computational Studies

#### Molecular Docking

*In silico* binding of synthesized compounds with respect to inhibition of human serine hydroxymethyltransferase 2 (SHMT2) protein was performed by molecular docking in the active site of the target protein using AutoDockTools 4.2.51 (Morris et al., 2009). The 3D crystal structure of human SHMT2 (PDB ID: 5V7I) was downloaded from the RCSB Protein Data Bank and used as the model for docking. The co-crystalized hetero molecules and water were removed from the target protein. The ligand structures (**4b** and **4c**) were constructed using ChemDraw ultra 19.0 software and subsequently converted to 3D structures and saved in .pdb format using Chem3D ultra 19.0 software. The ligand energies were minimized using MOPAC (semiempirical quantum mechanics) with AM1 MOZYME geometry acceleration with 100 iterations, and RMS gradient of 0.10. For each docked ligand, ten poses were generated. The structure with relative lower binding free energy (Kcal/mol) was selected as best conformation among all the poses. In order to validate the results, the co-crystal ligand 8Z1 ((4R)-6-amino-3-methyl-4-(propan-2-yl)-4-[3-(pyrrolidin-1-yl)-5-(trifluoromethyl)phenyl]-1,4-dihydropyrano[2,3-c]pyrazole-5-carbonitrile) was re-docked with the target protein. PyMol and LIGPLOT tools were used for 3D and 2D visualization of the docked complexes, respectively (Wallace et al., 1995).

#### Molecular Descriptors and Other Studies

Pharmacological and physiochemical properties of the synthesized molecules were evaluated by using Molinspiration and SwissADME online web tools (Daina et al., 2017). The lipophilicity (logP), topological polar surface area (TPSA), and drug likeliness were determined for all the synthesized compounds. To explore the properties of molecular flexibility, complexity, and shape index of the synthesized molecules, OSIRIS property explorer tool was used.

## Results and Discussions

### Chemical Synthesis

The synthesis of the novel pyran-linked phthalazinone-pyrazole hybrids was accomplished by employing an atom economical multicomponent reaction strategy. A phthalazinone derivative, 3-(1,4-dioxo-3,4-dihydrophthalazin-2(1H)-yl)-3-oxopropanenitrile **1**, with an appropriately substituted oxopropanenitrile group that involves in the multicomponent reaction was synthesized from phthalic anhydride and 2-cyanoacetohydrazide (Kumar et al., 2014). The other components employed in this MCR were substituted 1H-pyrazole-5-carbaldehyde **2a** and an active methylene–containing compounds. Initially, an optimization study for the one-pot reaction was undertaken with model substrate 1H-pyrazole-5-carbaldehyde **2a** along with other components such as phthalazinone **1** and malononitrile **3a** to identify the best reaction conditions (Table 1). In this study, different parameters such as base catalyst, solvents, reaction temperature, and catalyst loading were investigated. For this purpose, different organic bases such as L-proline, pyridine, and piperidine were screened as catalyst in solvents like methanol, ethanol, and dimethylformamide (DMF) at varying temperature. The model MCR with the tested base catalysts proceeded with modest yields (34–45%) using DMF as solvent at room temperature in 7.5–9 h (entries 7–9). Interestingly, on using protic solvents like ethanol and methanol, the yields of the reaction were improved (40–60%), accompanied by reduction in reaction time (4.5–5.5 h, entries 1–6). The results showed that both the protic solvents were equally efficient; however, L-proline provided the best yields in all the cases. Temperature is a critical parameter in optimization studies; therefore, we investigated the model reaction with L-proline at elevated temperature (70–75°C) with all the three base catalysts. It was observed that the reaction in DMF and methanol afforded 70 and 80% yield, respectively, in reaction time of 1.5 h (entries 11 and 12). However, the best results were obtained with ethanol and the reaction afforded the product **4a** in very good yield (88%) in a short reaction time of only 50 min (entry 10). To further improve the efficiency of the reaction, different catalyst loadings were also investigated; nonetheless, 20% catalyst loading showed the best results (Table 2).

**TABLE 1:** Optimization studies of the multicomponent reaction with the model substrate **2a**.

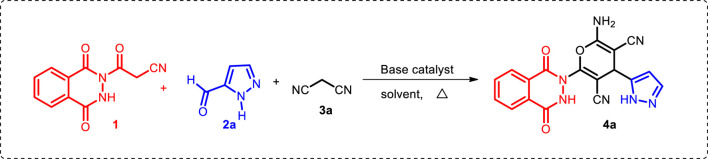					
Entry	Solvent	20 mol % catalyst	Temperature (^o^C)	Time (h)	4a (%)
1	Ethanol	L-proline	rt	5	60
2	Ethanol	Pyridine	rt	5	45
3	Ethanol	Piperidine	rt	4.5	40
4	Methanol	L-proline	rt	5	60
5	Methanol	Pyridine	rt	5.5	45
6	Methanol	Piperidine	rt	5	42
7	DMF	L-proline	rt	7.5	45
8	DMF	Pyridine	rt	9	35
9	DMF	Piperidine	rt	8	34
10	Ethanol	L-proline	70–75	50 min	88
11	Methanol	L-proline	70–75	1.5	80
12	DMF	L-proline	70–75	1.5	70

**TABLE 2:** Effect of catalyst loading.

Entry	Amount of catalyst	Time (min)	4a (%)
1	10 mol % L-proline	90	82
2	20 mol % L-proline	50	88
3	30 mol % L-proline	45	81

**SCHEME 1:**
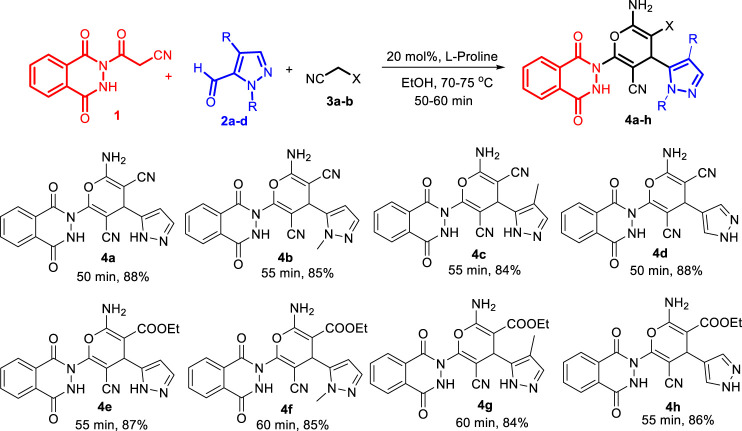
One-pot synthesis of novel pyran-linked phthalazinone-pyrazole hybrids **4a**–**h**.

**FIGURE 2:**
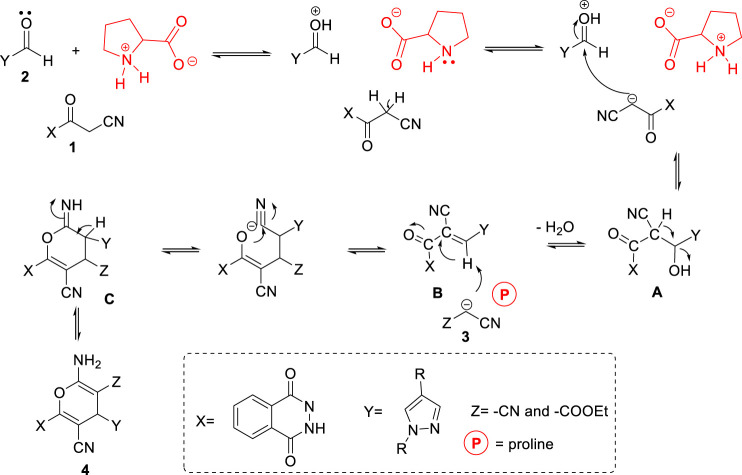
Plausible mechanism of formation of pyran-linked phthalazinone-pyrazole hybrids.

Finally, the scope of the reaction was explored under the optimized conditions to afford a series of novel pyran-linked phthalazinone-pyrazole hybrids. The MCR was carried out with phthalazinone derivative **1**, various substituted 1H-pyrazole-5-carbaldehydes **2a-d** and active methylene–containing compounds (malononitrile **3a** and ethyl 2-cyanoacetate **3b**) with L-proline as a catalyst in ethanol at elevated temperature (Scheme 1). The multicomponent reaction proceeded with similar efficiency with different reactants, and the desired novel pyran-linked phthalazinone-pyrazole hybrids **4a**–**h** were obtained in high yield in the range of 84–88%. A plausible mechanism for the synthesis of hybrids **4a**–**h** in the presence of L-proline is proposed (Figure 2). L-proline exists in the form of zwitterion acting as a bifunctional catalyst. It protonates the aldehyde **2** and also abstracts the hydrogen of active methylene functionality of **3** to afford carbanion species, which further attacks the protonated aldehyde **2** forming intermediate **A**. This is followed by loss of water molecule from **A,** affording an α,β-unsaturated diketone intermediate **B**. The intermediate B undergoes Michael addition by carbanion **3** followed by nucleophilic addition to afford imine intermediate **C**. Finally, the tautomerization of intermediate **C** yields the final product **4**.

### Anticancer Activity

The phthalazine and pyrazole moieties are known to exhibit anticancer activity; therefore, the cytotoxicity potential of the synthesized novel pyran-linked phthalazinone-pyrazole hybrids was investigated. Globally, lung cancer is a leading cause of cancer death, and on the other hand, cervical cancer is one of the major cancers in women with high incidence and mortality (Thakur et al., 2020; Ferlay et al., 2021). Therefore, we screened the synthesized novel hybrids (**4a**–**h**) against human lung carcinoma cells (A549) and human cervical carcinoma cells (HeLa) by employing the MTT assay. All the tested hybrids were active and displayed moderate-to-good activity with an IC_50_ ranging from 9.8 to 41.6 µM against A549 cells and 10.1 to 31.6 µM against HeLa cells (Table 3). Based on the results, compound **4c** was the most promising hybrid from the series that exhibited significant activity against A549 cells with an IC_50_ value of 9.8 µM. In case of HeLa cancer cell lines, it exhibited similar potency with IC_50_ value of 10.1 µM. The other hybrids **4a**, **4b**, **4f**, and **4g** showed good activity against A549 cells with IC_50_ values of 14.1, 10.6, 16.4, and 15.6 µM, respectively, and modest cytotoxicity against HeLa cells with IC_50_ values of 17.9, 11.8, 18.6, and 13.1 µM, respectively. The hybrids **4d**, **4e,** and **4h** were the least active compounds in the series against the tested cells. The structure activity relationship (SAR) pattern of the novel pyran-linked phthalazinone-pyrazole hybrids is provided as an infographic in the table.

**TABLE 3:** Cytotoxicity and structure activity relationship of novel pyran-linked phthalazinone-pyrazole hybrids **4a–h**.

Compound	IC_50_ (µM ± SD)	
	A549	HeLa
**4a**	14.1 ± 0.9	17.9 ± 1.0
**4b**	10.6 ± 1.2	11.8 ± 1.2
**4c**	9.8 ± 0.9	10.1 ± 0.9
**4d**	28.9 ± 1.3	30.5 ± 1.4
**4e**	26.3 ± 1.2	20.9 ± 1.1
**4f**	16.4 ± 1.0	18.6 ± 0.9
**4g**	15.6 ± 1.1	13.1 ± 0.8
**4h**	41.6 ± 1.8	31.6 ± 1.3
**Doxorubicin (positive control)**	0.69 ± 0.1	0.81 ± 0.1
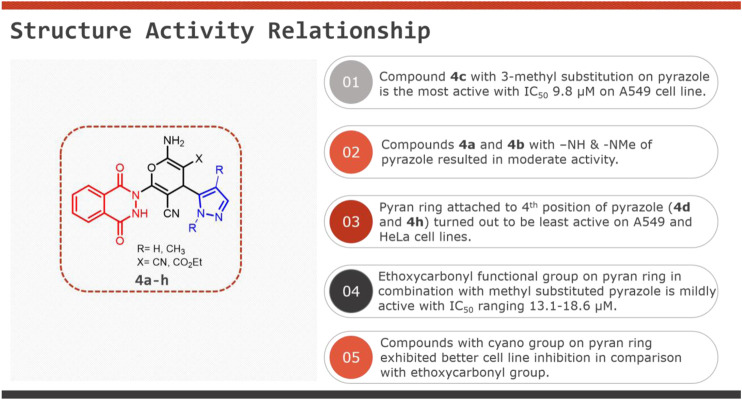

### *In Silico* Binding Studies

**FIGURE 3:**
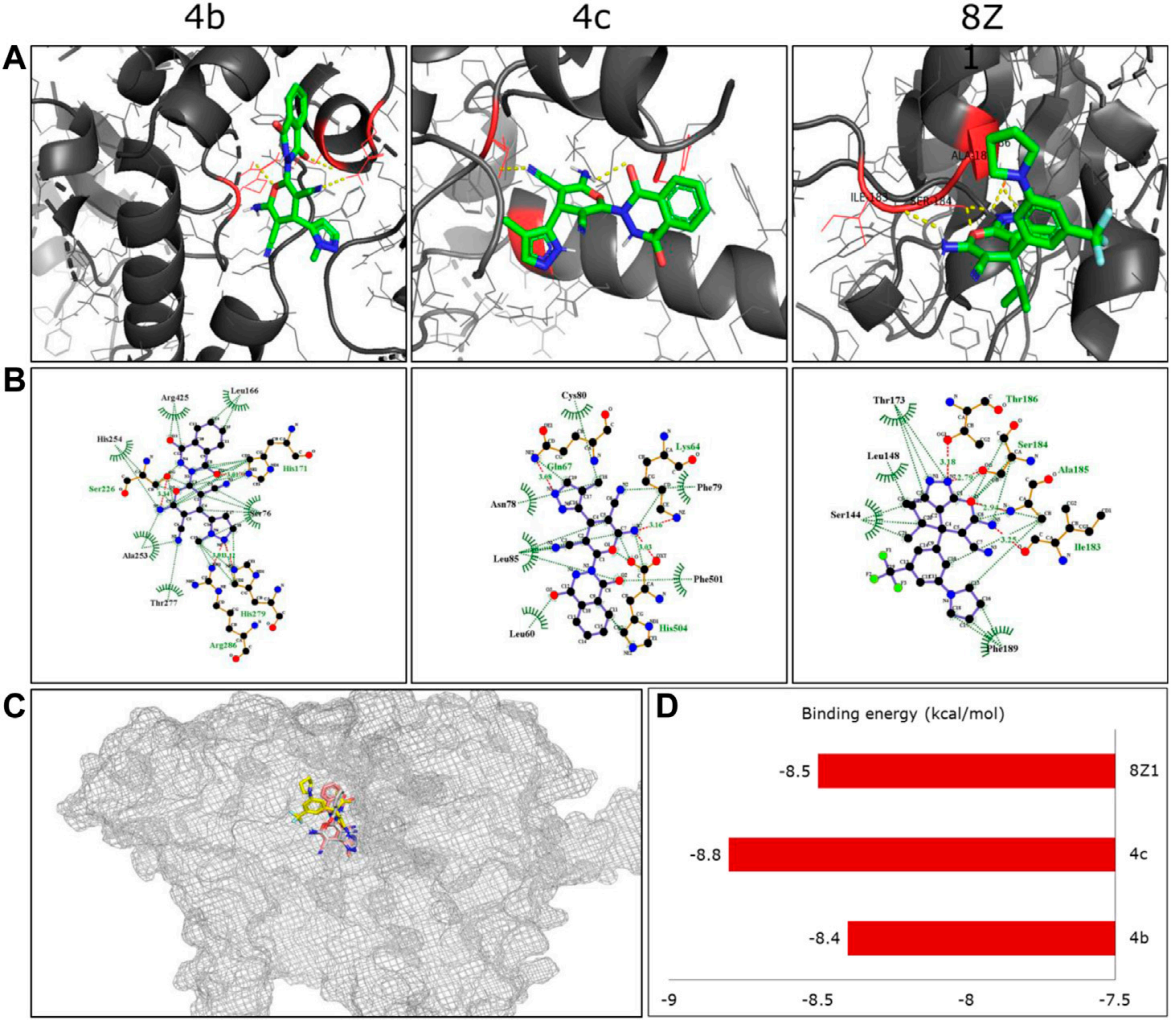
**(A)** Three-dimensional binding conformations of the synthesized molecules against human serine hydroxymethyltransferase 2 protein (PDB ID: 5V7I). The yellow color dotted lines represent the hydrogen bonds as well as polar interactions. **(B)** Two-dimensional binding interactions of the docked ligand molecules with target protein. **(C)** Overlapped structures of the synthesized compounds along with the co-crystalized ligand in the protein active site. **(D)** The binding energies (kcal/mol) of the docked compounds with target protein.

The serine hydroxymethyltransferase (SHMT) catalyzed the conversion of serine to glycine, thereby releasing one carbon unit essential in cell regulation. One of the mitochondrial isoforms, SHMT2, is linked to cancer survival and is upregulated in the lung and other cancer cells (Tong et al., 2020). A pyrazolopyran scaffold (8Z1) is reported to inhibit this protein at the molecular level. The structural similarity of our novel pyran-linked phthalazinone-pyrazole hybrids to 8ZI prompted us to perform molecular docking studies to understand the binding affinity with SHMT2 protein. From the preliminary anticancer results, the active compounds **4b** and **4c** were examined for docking with hydroxymethyltransferase 2 protein co-crystalized with 8Z1. The ligands exhibited good binding affinities toward target protein as compared with 8Z1. The hydrogen and hydrophobic interactions played a major role in the binding of ligands with target protein. Almost ten different conformations per each docked ligand were generated, and the best conformation was displayed in the Figures 3A,B. The docking results revealed that the hybrids **4b** and **4c** occupied the same binding site of the co-crystalized ligand binding site (Figure 3C) and exhibited excellent affinity with binding energy of −8.4 and −8.8 kcal/mol, respectively (Figure 3D). Interestingly, the binding affinity of hybrid **4c** was better than that of the co-crystal ligand 8Z1 (a pyrazolopyran inhibitor), which exhibited −8.5 kcal/mol of binding energy. The docking results influenced both hydrogen and hydrophobic interactions, and the amino acid residues of the target protein that interacted with the docked ligands are presented in Table 4.

**TABLE 4:** Amino acid residues of the target protein that interacts with ligands **4a** and **4b**.

Ligand	Protein–ligand interactions	
	H-bond/s	Hydrophobic bonds
**4b**	His171, Ser226, His279, Arg286	Ser76, Leu166, Ala253, His254, Thr277, Arg425
**4c**	Lys64, Gln67, His504	Leu60, Asn78, Phe79, Cys80, Leu85, Phe501
8Z1 (co-crystal ligand)	Thr186, Ser184, Ala185, Ile183	Ser144, Leu148, Thr173, Phe189

### Drug Likeliness Nature

**FIGURE 4:**
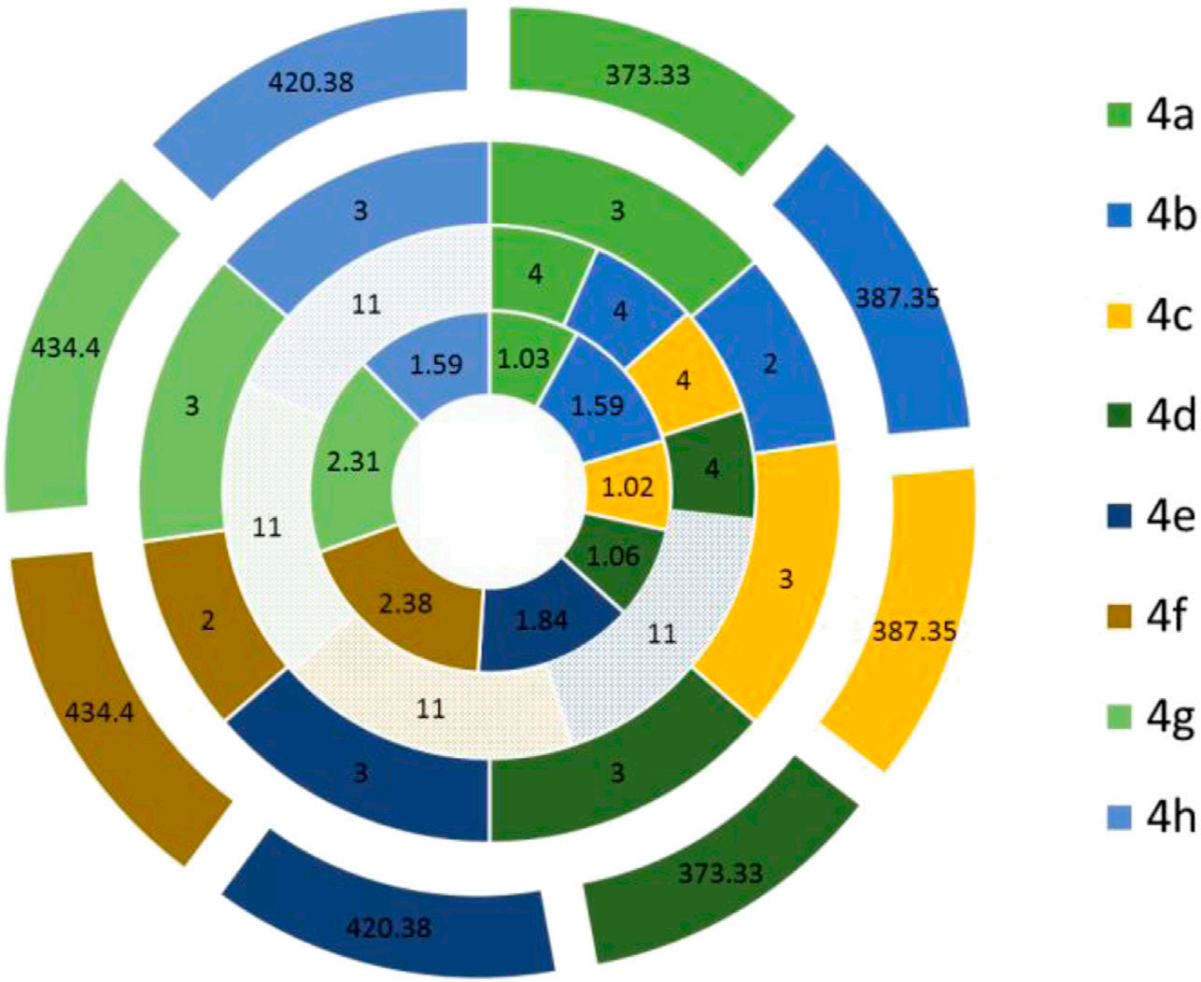
Predicted parameters of Lipinski’s rule of five for the synthesized compounds were calculated using SwissADME and represented in the Doughnut graph. The compounds that violate the rule were represented in the filled pattern format. Each circle represents one parameter of Lipinski’s rule. The parameters LogP, HBA, HBD, and MW showed from inner to outer circles, respectively.

**FIGURE 5:**
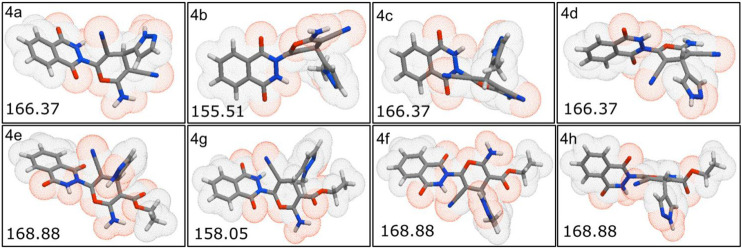
Three-dimensional polar surface area visualization of all the synthesized molecules using Molinspiration tool.

The physiochemical properties of all the synthesized molecules toward drug likeliness were calculated, and the results revealed that all the compounds showed acceptable properties. Based on Lipinski’s rule of five and its components, the endpoint values for all the four parameters that describe possible outcome of a drug candidate for good absorption are MW ≤ 500, LogP ≤ 5, HBD ≤ 10, and HBA ≤ 5 (Lipinski, 2004). It was observed that almost all the molecules occupied space within Lipinski’s rule of five (Figure 4); besides, only four compounds exhibited only a single violation. Compounds **4e**, **4f**, **4g,** and **4h** showed one violation with respect to the number of hydrogen bond donors (HBDs). The predicted LogP values for all the compounds indicate that compounds exhibit good permeability across cell membrane. The calculated LogP values for the test compounds are in the range of 1.03–2.38, which are in the accepted range. The 3D polar surface area visualizations were generated, and the calculated TPSA was >140 Å for all the compounds; this indicates the reduced oral bio-availability of the compounds (Figure 5; Prasanna and Doerksen (2009)).

### Molecular Flexibility, Complexity, and Shape Index

**FIGURE 6:**
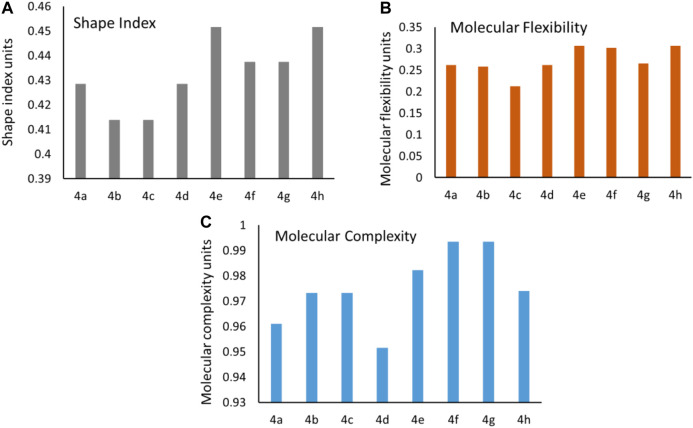
Predicted values of the **(A)** shape index, **(B)** molecular flexibility, and **(C)** molecular complexity for the synthesized molecules.

The conformational flexibility, molecular complexity, and shape index of a molecule are important parameters influencing the ligand to target protein binding in drug design (Wicker and Cooper, 2016; Méndez-Lucio and Medina-Franco, 2017). Shape index of the synthesized molecules was calculated using DataWarrior, and it determines 3D shape of compounds. In general, shape index less than 0.5 in molecules suggests the presence of spherical or non-flat scaffolds, whereas shape index more than 0.5 is for flat scaffolds. The synthesized molecules exhibited an average shape index of 0.43, which is less than 0.5 [Figure 6A]. These results suggest that the compounds were spherical or non-flat scaffolds. Few reports proposed that a majority of non-flat or spherical compounds were observed in natural products (Lovering et al., 2009). This suggests that spherical scaffolds may be essential for anticancer activity. Further, the structural flexibility and complexity of the molecules were predicted using DataWarrior. Less than 0.5 value of flexibility and complexity in molecules suggests that the molecules are of low complexity and flexibility. The synthesized molecules exhibited an average of 0.27; this indicates the molecules are low-to-intermediate flexible [Figure 6B]. Additionally, 0.97 average value for molecular complexity in molecules suggests the molecules are complex in nature [Figure 6C] (Méndez-Lucio and Medina-Franco, 2017).

## Conclusion

In conclusion, a series of novel pyran-linked phthalazinone-pyrazole hybrids were designed and synthesized by a facile one-pot three-component reaction in the presence of L-proline, which acted as a catalyst. The assessment of cytotoxicity potency revealed that the methyl substitution on pyrazole and two cyano groups on pyran, as in **4b** and **4c**, were necessary to elicit a good inhibitory response toward the tested lung and cervix cancer cells. Interestingly, the molecular modeling studies with hydroxymethyltransferase 2 (SHMT2), a protein that is upregulated in lung and other cancers, revealed that the active hybrids **4b** and **4c** displayed comparable-to-superior binding affinity than ligand 8Z1, a pyrazolopyran inhibitor. The hybrids showed good drug likeliness properties as elucidated by Lipinski’s rule, with four compounds exhibiting a single violation. Similarly, the results obtained through molecular descriptor predictions provided an understanding about the potential of the synthesized molecules to transform into lead-like molecules. It can be said that upon further structural optimizations (particularly **4b** and **4c**) and a thorough biological mechanistic investigation, the synthesized scaffolds may likely turn into potential anticancer agents.

## Data Availability

The original contributions presented in the study are included in the article/Supplementary Material; further inquiries can be directed to the corresponding authors.
