# Supplementary material for: Novel Pyran-Linked Phthalazinone-Pyrazole Hybrids: Synthesis, Cytotoxicity Evaluation, Molecular Modeling, and Descriptor Studies
Source: Front Chem. 2021 May 24;9:666573. doi: 10.3389/fchem.2021.666573 (PMC8181751; doi:10.3389/fchem.2021.666573)
Supplement: Supplementary file 1 [file DataSheet1.PDF]

Novel pyran linked phthalazinone-pyrazole hybrids: Synthesis, cytotoxicity evaluation, molecular modelling and descriptors studies

M. Shaheer Malik[1]*, Basim H. Asghar[1]*, Riyaz Syed[2], Reem I. Alsantali[3], Moataz Morad[1], Hatem M. Altass[1,4], Ziad Moussa[5], Ismail I. Althagafi[1], Rabab S. Jassas[6], Saleh A. Ahmed[1,4,7]*

Supporting information

- Spectra of selected novel pyran linked phthalazinone-pyrazole hybrids

1H NMR IN DMSO-D6
AV 400MHz

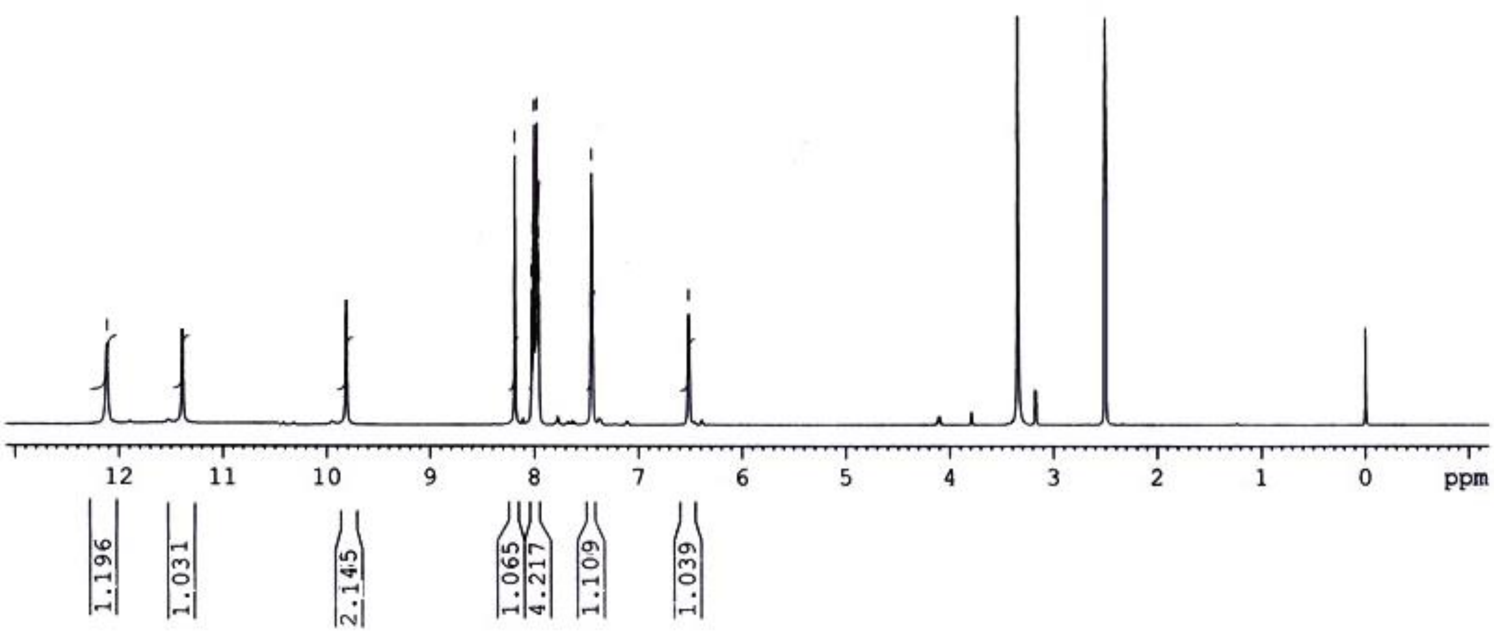

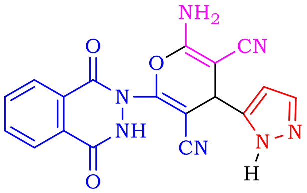

4a

Fig. 1: ^{1}H NMR spectrum of compound 4a

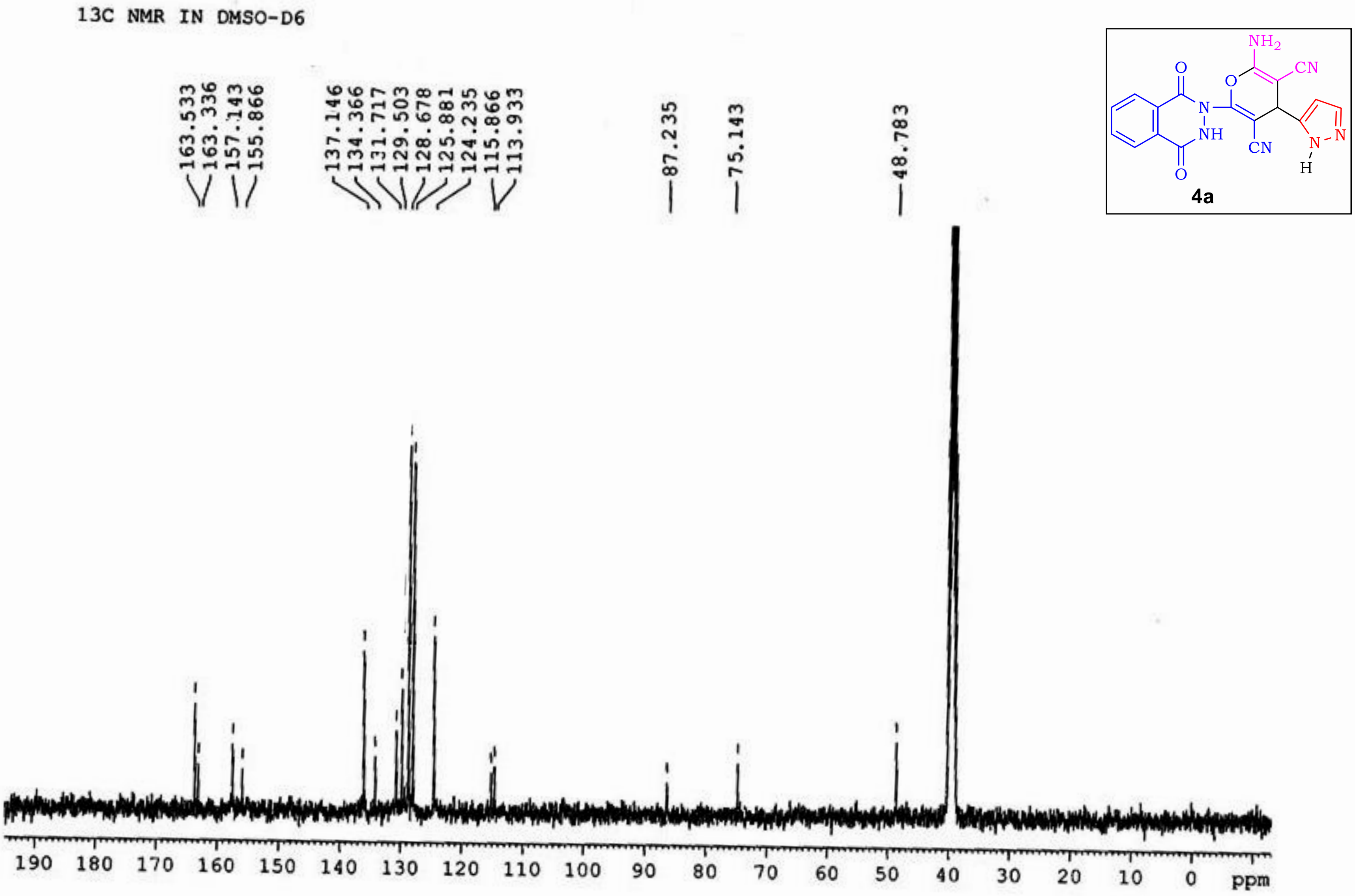

Fig. 2: ^{13}C NMR spectrum of compound 4a

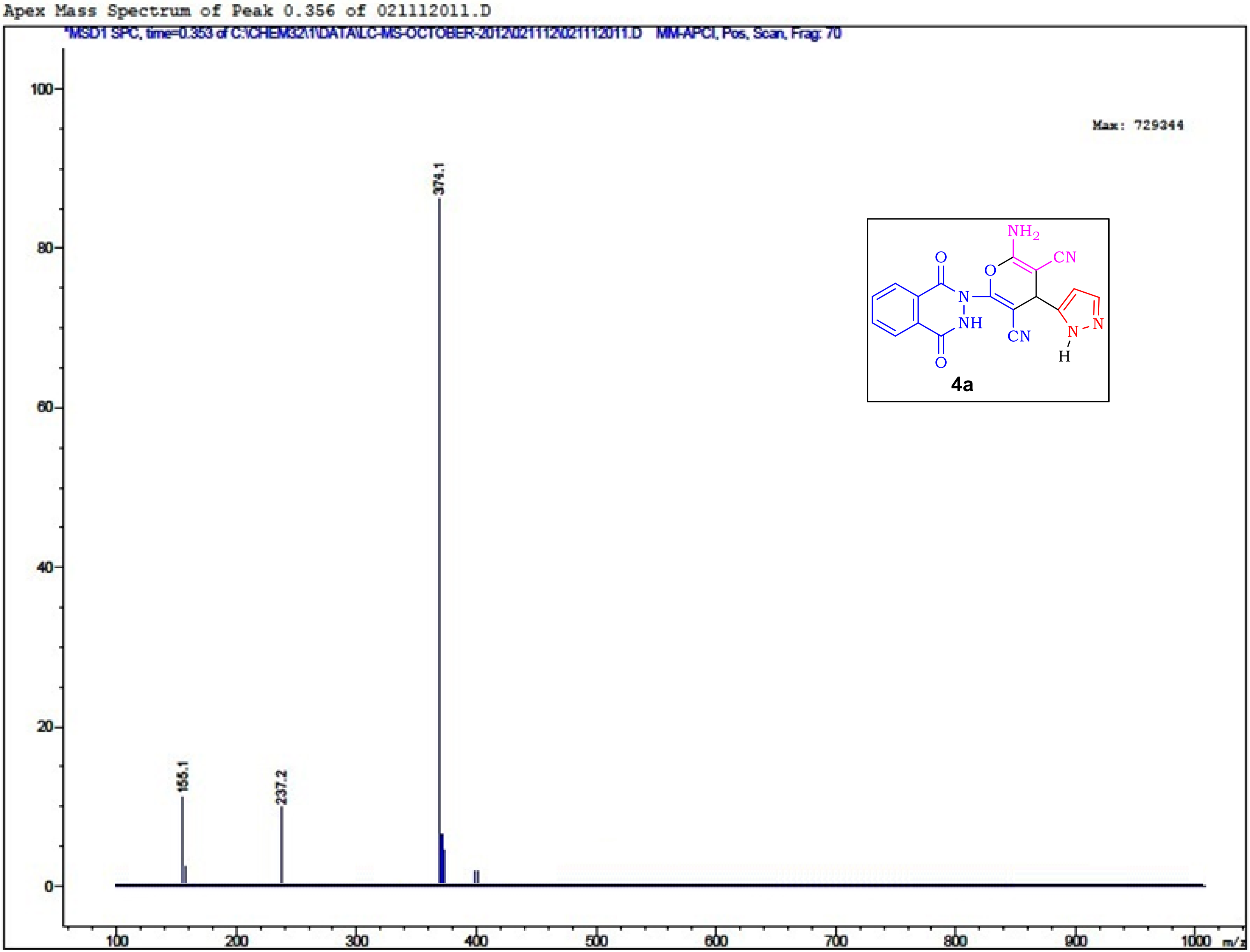

Fig. 3: Mass spectrum of compound 4a

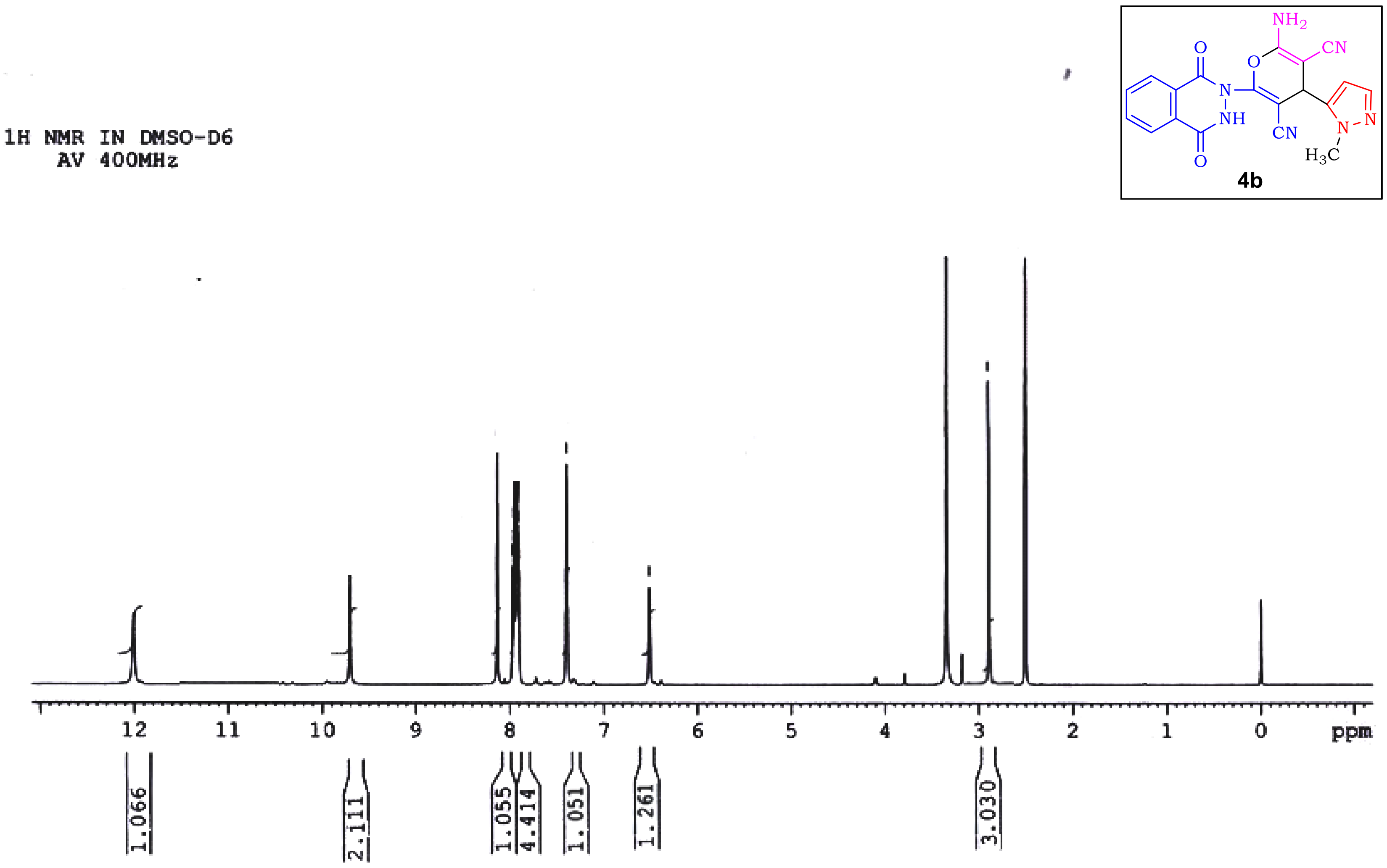

Fig. 4: ^{1}H NMR spectrum of compound 4b

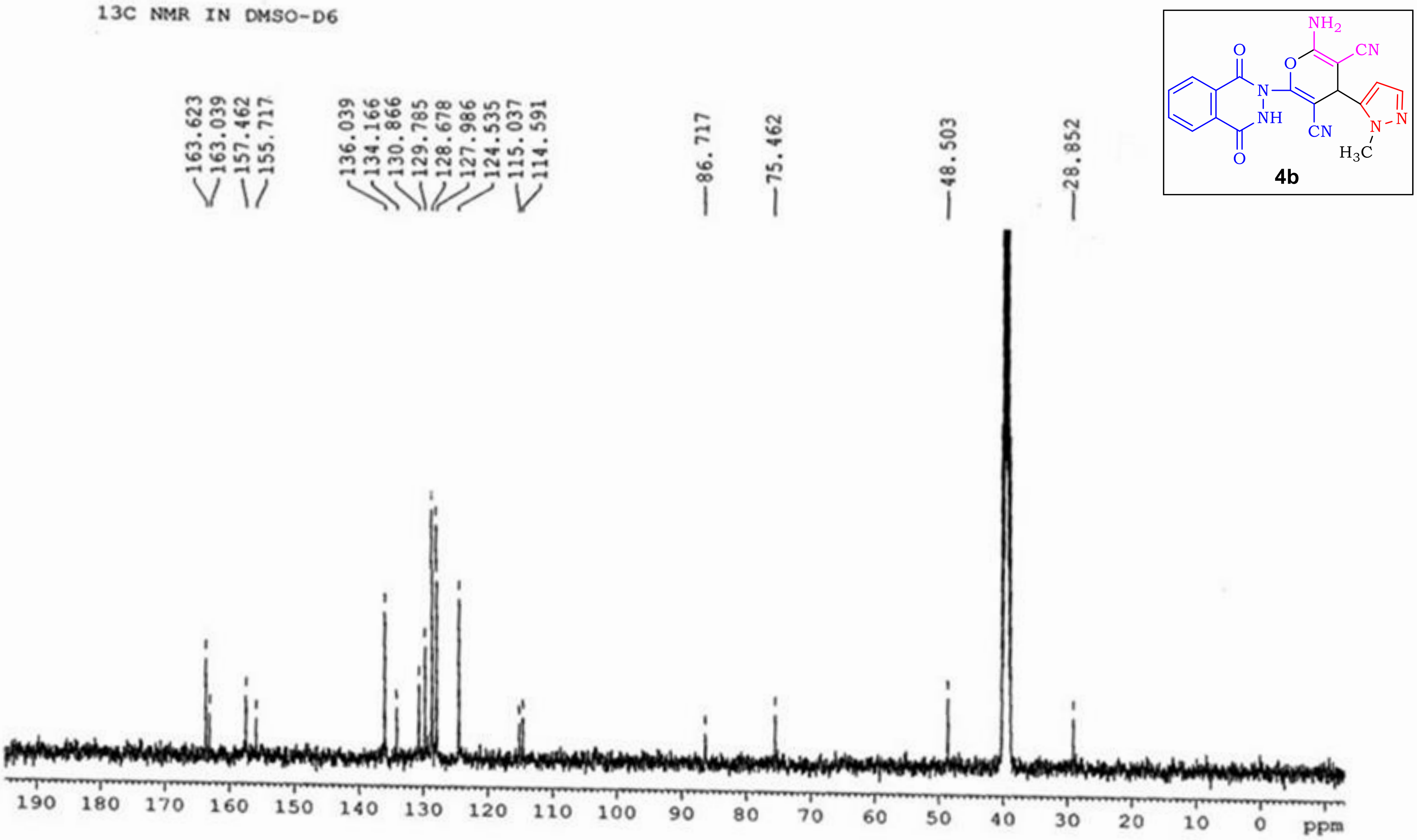

Fig. 5: ^{13}C NMR spectrum of compound 4b

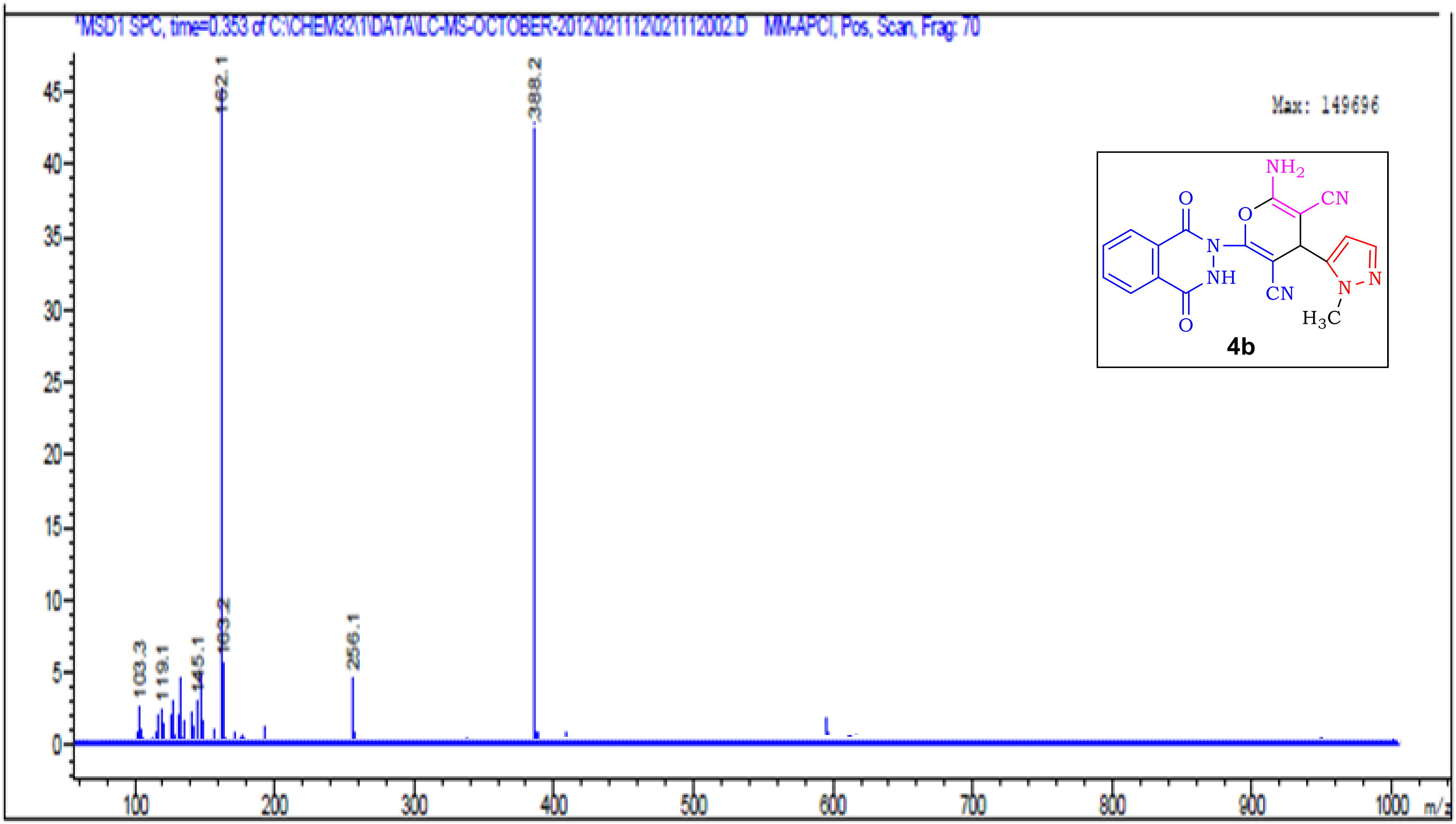

Fig. 6: Mass spectrum of compound 4b

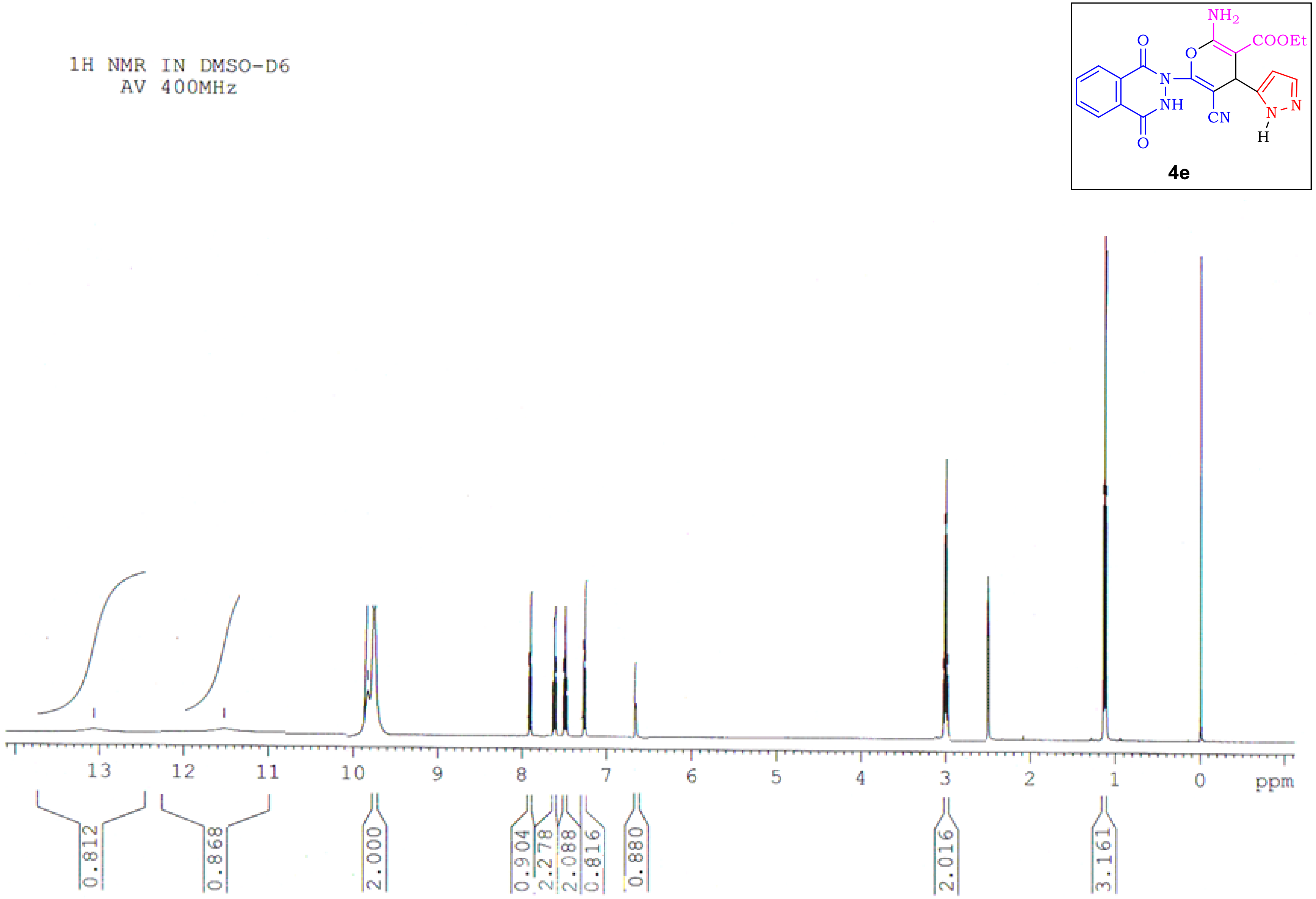

Fig. 7: ^{1}H NMR spectrum of compound 4e

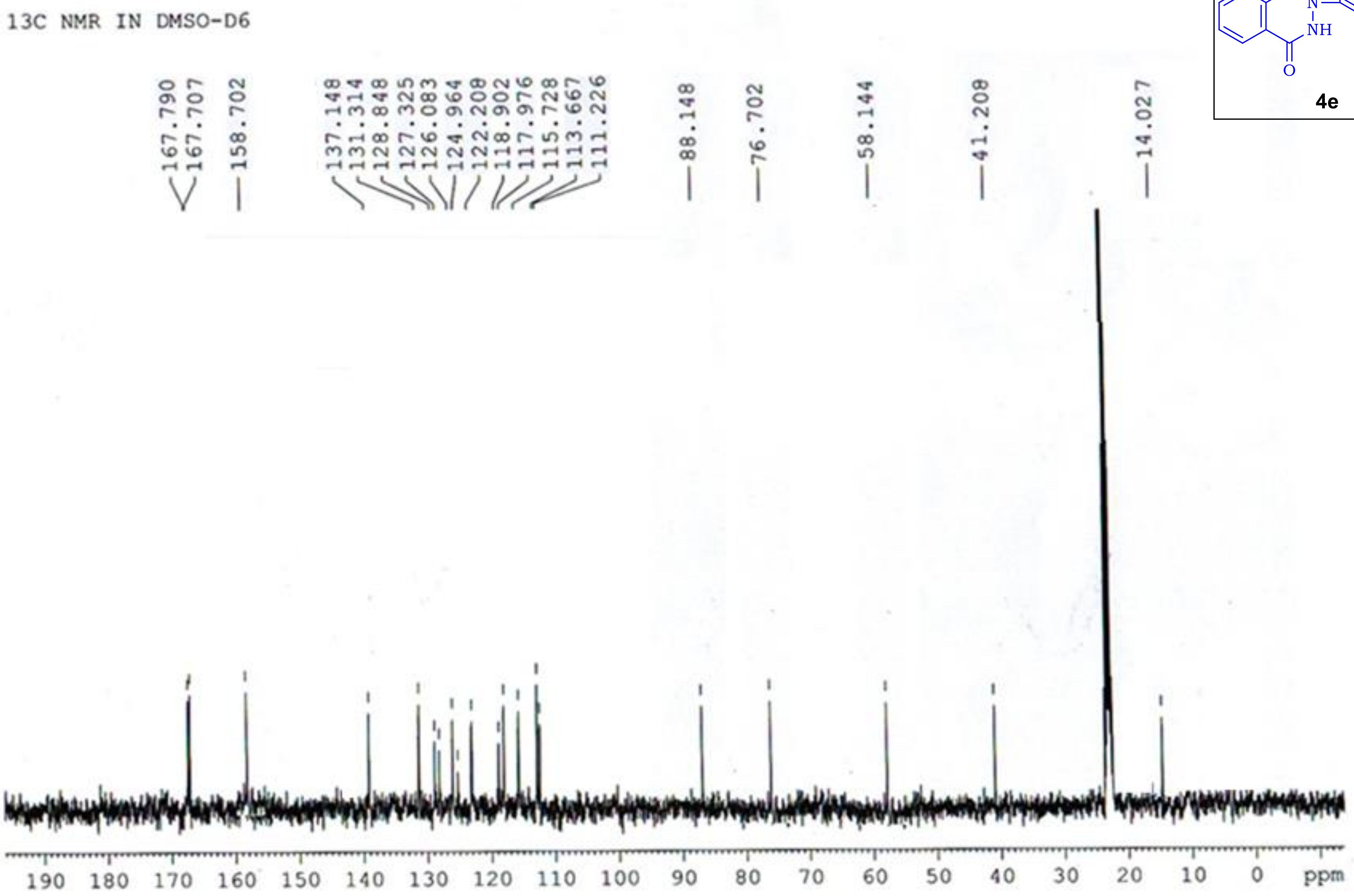

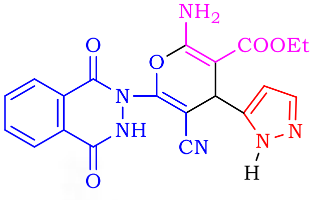

4e

Fig. 8: ^{13}C NMR spectrum of compound 4e

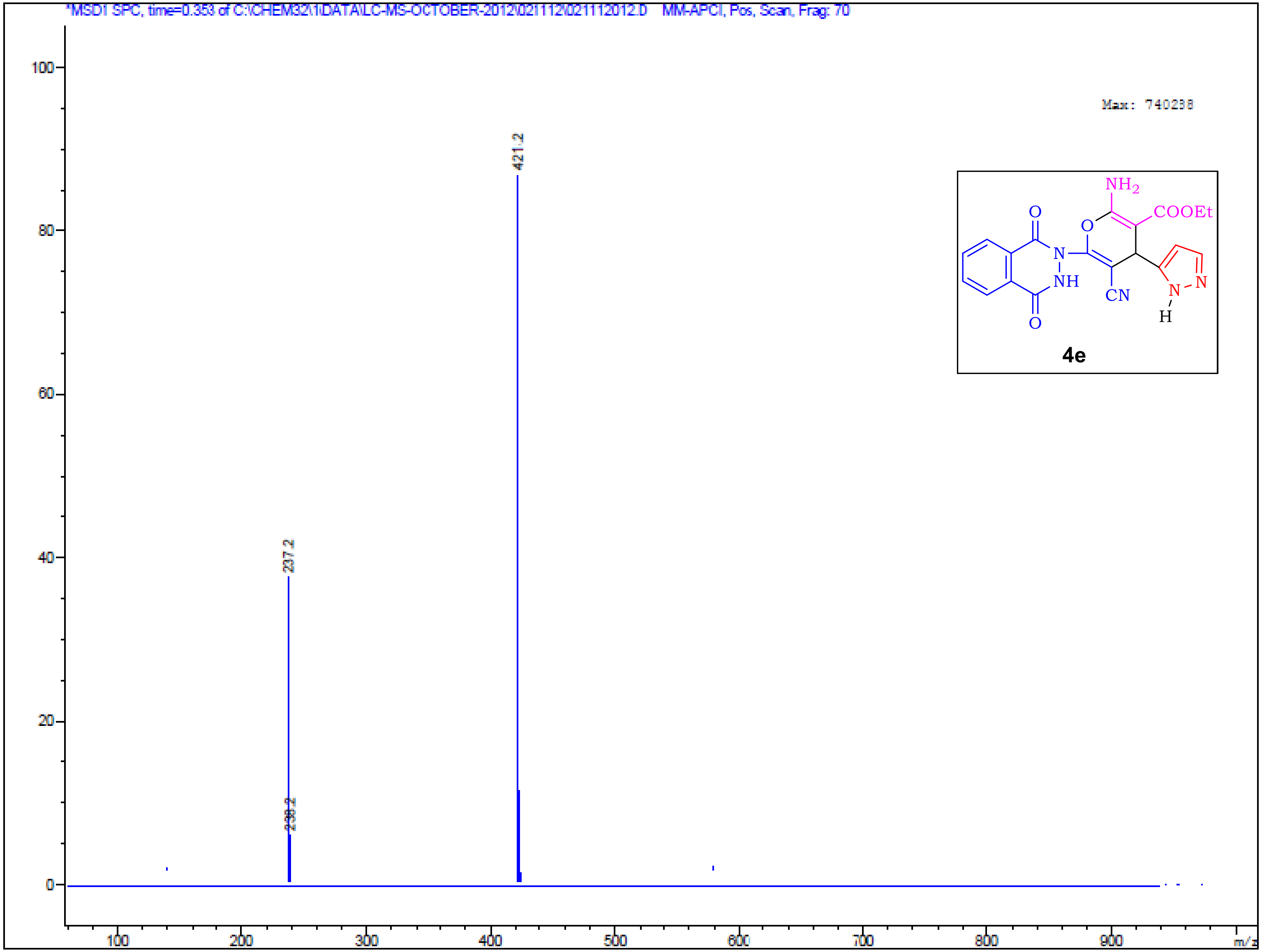

Fig. 9: Mass spectrum of compound 4e

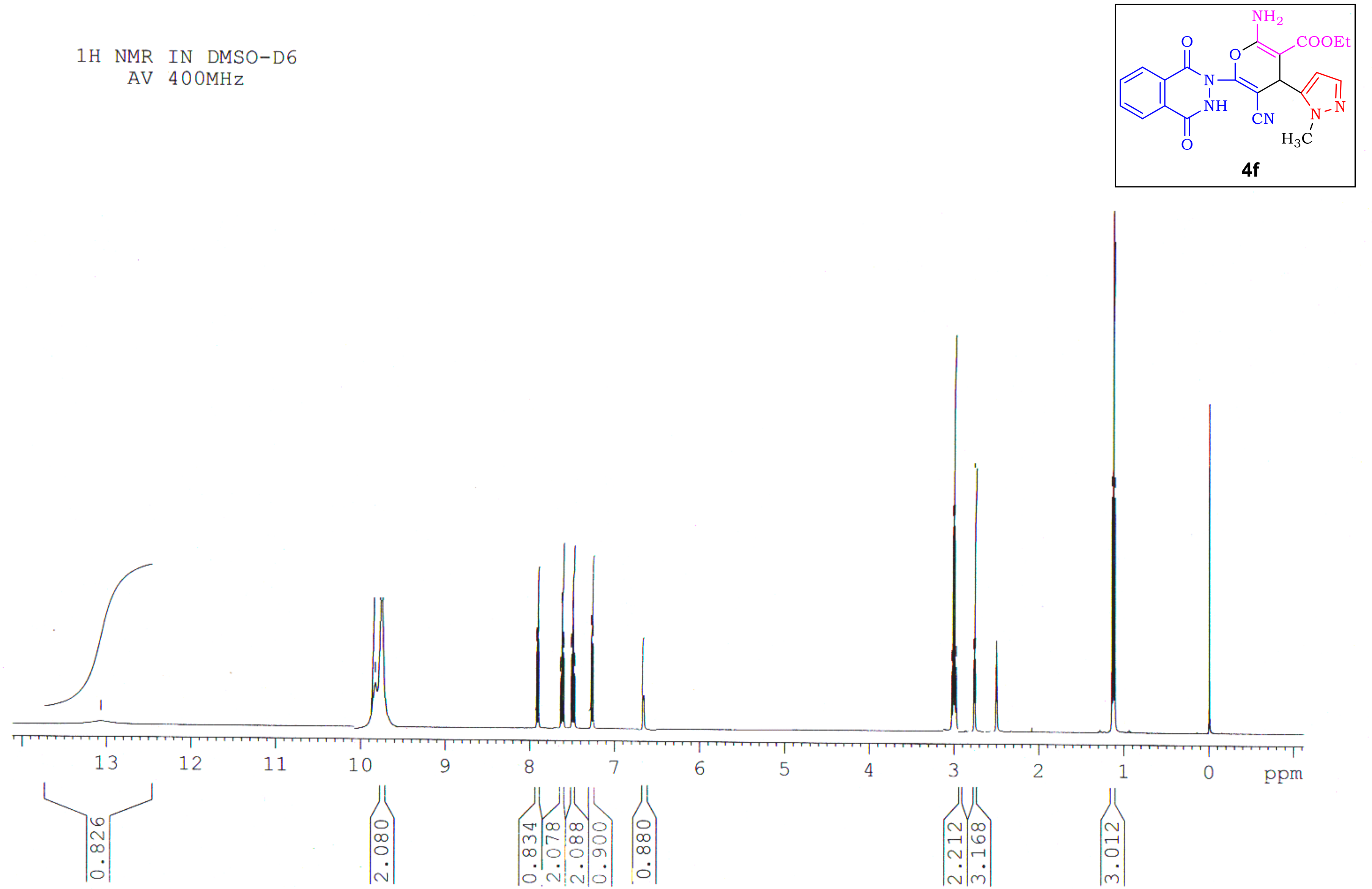

Fig. 10: ¹H NMR spectrum of compound 4f

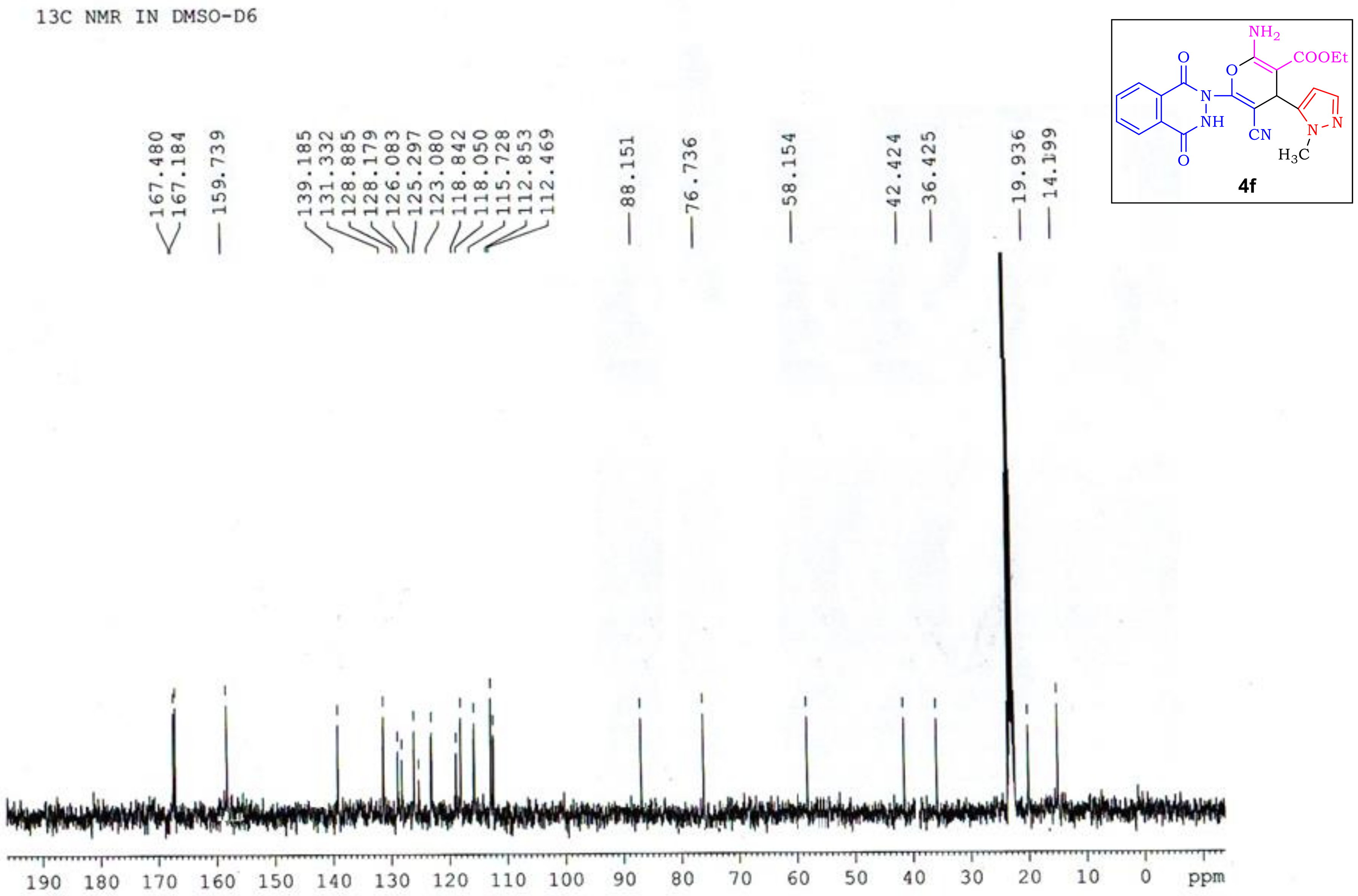

Fig. 11: ^{13}C NMR spectrum of compound 4f

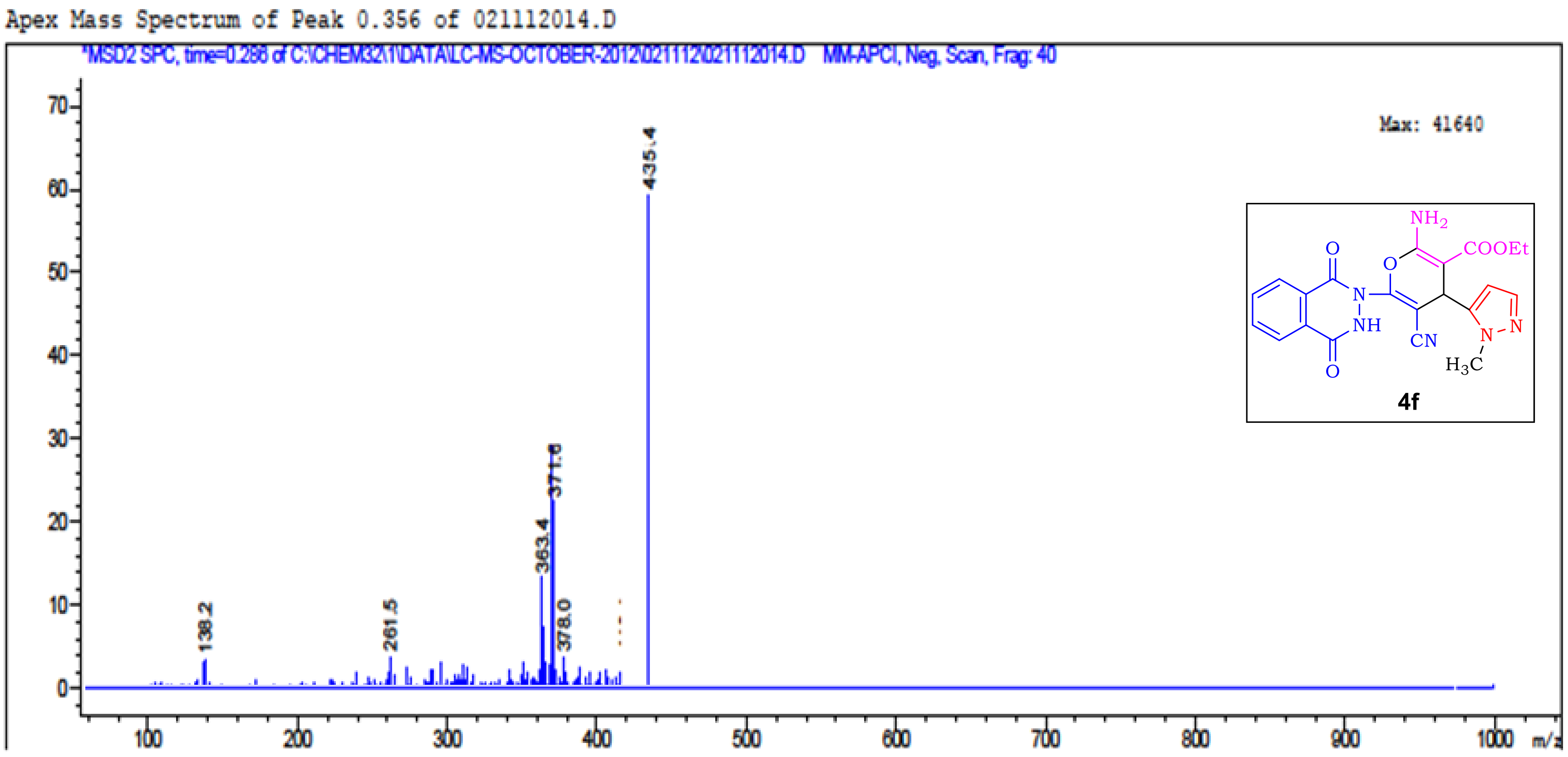

Fig. 12: Mass spectrum of compound 4f
